# Common and Distinctive Functions of the Hippo Effectors Taz and Yap in Skeletal Muscle Stem Cell Function

**DOI:** 10.1002/stem.2652

**Published:** 2017-06-27

**Authors:** Congshan Sun, Vanessa De Mello, Abdalla Mohamed, Huascar P. Ortuste Quiroga, Amaya Garcia‐Munoz, Abdullah Al Bloshi, Annie M. Tremblay, Alexander von Kriegsheim, Elaina Collie‐Duguid, Neil Vargesson, David Matallanas, Henning Wackerhage, Peter S. Zammit

**Affiliations:** ^1^ Randall Division of Cell and Molecular Biophysics King's College London London UK; ^2^ School of Medicine, Medical Sciences & Nutrition University of Aberdeen Foresterhill Aberdeen Scotland, UK; ^3^ Systems Biology Ireland, Conway Institute Dublin Ireland; ^4^ Stem Cell Program, Children's Hospital Boston Massachusetts USA; ^5^ Department of Stem Cell and Regenerative Biology Harvard University Cambridge Massachusetts USA; ^6^ Harvard Stem Cell Institute Cambridge Massachusetts USA; ^7^ Centre for Genome Enabled Biology and Medicine, School of Medicine, Medical Sciences and Nutrition University of Aberdeen Foresterhill Aberdeen Scotland, UK; ^8^ Faculty of Sport and Health Sciences Technical University of Munich Munich Germany

**Keywords:** Taz, Yap, Tead, Satellite cells, Muscle stem cells

## Abstract

Hippo pathway downstream effectors Yap and Taz play key roles in cell proliferation and regeneration, regulating gene expression especially via Tead transcription factors. To investigate their role in skeletal muscle stem cells, we analyzed Taz in vivo and ex vivo in comparison with Yap. Small interfering RNA knockdown or retroviral‐mediated expression of wild‐type human or constitutively active TAZ mutants in satellite cells showed that TAZ promoted proliferation, a function shared with YAP. However, at later stages of myogenesis, TAZ also enhanced myogenic differentiation of myoblasts, whereas YAP inhibits such differentiation. Functionally, while muscle growth was mildly affected in Taz (gene *Wwtr1* ^–/–^) knockout mice, there were no overt effects on regeneration. Conversely, conditional knockout of Yap in satellite cells of *Pax7^Cre‐ERT2/+^: Yap^fl^°^x/fl^°^x^:Rosa26^Lacz^* mice produced a regeneration deficit. To identify potential mechanisms, microarray analysis showed many common TAZ/YAP target genes, but TAZ also regulates some genes independently of YAP, including myogenic genes such as *Pax7*, *Myf5*, and *Myod1* (ArrayExpress–E‐MTAB‐5395). Proteomic analysis revealed many novel binding partners of TAZ/YAP in myogenic cells, but TAZ also interacts with proteins distinct from YAP that are often involved in myogenesis and aspects of cytoskeleton organization (ProteomeXchange–PXD005751). Neither TAZ nor YAP bind members of the Wnt destruction complex but both regulated expression of Wnt and Wnt‐cross talking genes with known roles in myogenesis. Finally, TAZ operates through Tead4 to enhance myogenic differentiation. In summary, Taz and Yap have overlapping functions in promoting myoblast proliferation but Taz then switches to enhance myogenic differentiation. Stem Cells *2017;35:1958–1972*

**Significance Statement**

Hippo pathway effectors Yap and Taz play key roles in cell proliferation and tissue growth. We analyzed Taz in comparison with Yap in muscle stem cells. Taz promoted proliferation, a function shared with Yap. However, Taz also enhanced differentiation into myotubes, unlike Yap. Muscle growth was affected in Taz (*Wwtr1^–/–^*) knockout mice, while satellite cell‐specific conditional knockout of Yap produced a regeneration deficit. Taz regulates some genes independently of Yap, and Taz also interacts with proteins distinct from Yap, mainly involved in myogenesis/cytoskeleton. In particular, Taz operates through Tead4 to enhance differentiation. In summary, Taz and Yap have overlapping functions in promoting myoblast proliferation but later, Taz also enhances myogenic differentiation.

## Introduction

Transcriptional cofactors Yap (*Yap1*) and Taz (*Wwtr1*) mainly regulate gene expression by binding Tead1–4 transcription factors. Together Yap, Taz, and Teads are the nexus of the Hippo signal transduction network that includes the Hippo kinase cascade, comprising kinases Mst1 (*Stk4*), Mst2 (*Stk3*), Lats1, and Lats2 1, 2, 3. Many other signaling modules also regulate Yap and Taz activity, such as mechanotransduction 4, glucose‐signaling 5, G‐protein coupled receptors (GPCR) 6, Wnt 7, 8, 9, Smad 10, 11, Notch 12, Pten‐Akt‐mechanistic target of rapamycin (mTOR) 13, 14, 15, 16, and Lkb1‐Ampk 17, 18, 19, 20. Yap and Taz are inhibited by phosphorylation of multiple HXRSS motifs by Lats1/2, which promotes localization to the cytosol, 14–3‐3 binding, and degradation 3, 21. Yap Ser127 and Taz Ser89 are key phosphorylation sites, and the mutation of Ser127 or Ser89 to alanine in Yap S127A and Taz S89A respectively prevents phosphorylation at these residues and results in constitutive activity.

Signaling modules involving Yap/Taz control skeletal muscle myogenesis and adaptation to exercise. Tead transcription factors bind CATTCC/GGAATG (MCAT or GTIIC motifs) found in/near promoters and enhancers of cardiac and skeletal muscle genes 3, 22, 23, 24. However, Tead1 binds muscle genes repressed in rhabdomyosarcoma 25, consistent with observation that Yap and Taz can also repress gene expression 26.

Satellite cells are responsible for postnatal skeletal muscle growth, hypertrophy, and repair/regeneration 27, and we have shown that Yap promotes proliferation of myoblasts 28, 29 but inhibits myogenic differentiation. YAP1 S127A expression in activated, but not quiescent, satellite cells causes embryonal rhabdomyosarcoma‐like tumours with short latency and 100% penetrance 25. Expression of YAP1 S127A in muscle fibers causes myopathy 30, whereas other types of Yap delivery or Yap mutants can cause hypertrophy 31, 32, with outcome likely dependent on Yap levels.

Taz harbors the same functionally important WW and Tead‐binding domains as Yap and often acts as a paralogue, but not always 2, 3. This is demonstrated by Yap or Taz knockout mice: while Yap knockout causes early embryonic lethality 33, 50% of Taz‐knockout (*Wwtr1^–/–^*) mice are viable but develop glomerulocystic kidney disease 34. Earlier reports suggest that Yap and Taz also have divergent functions in the skeletal muscle lineage: both Yap and Taz promote skeletal muscle fiber hypertrophy and regeneration 35, 36, but only Taz promotes fusion into multinucleated myotubes 29, 37, 38.

The distinct functions of YAP and TAZ are poorly understood, so we investigated their regulation, function, target genes, and binding partners in murine myoblasts. Taz and Yap have overlapping functions in promoting satellite cell proliferation. However, later in myogenesis, Taz enhances myogenic differentiation and fusion, a function not shared by Yap. Taz‐knockout (*Wwtr1* ^–/–^) mice had no overt muscle regeneration phenotype, unlike mice with conditional knockout of Yap in satellite cells. To understand mechanism, we examined gene expression, and found, for example, that YAP and TAZ both alter expression of multiple Wnt pathway genes. In addition to common targets, TAZ also regulates a separate set of genes from YAP during myogenesis, including myogenic factors *Pax7, Myf5,* and *MyoD*. Using proteomics to identify binding partners of TAZ and YAP, we discovered both common and distinct interactors, with TAZ binding proteins that regulate myogenesis and aspects of cytoskeleton organization. Finally, we found that TAZ operates through Tead4 in myoblasts to enhance myogenic differentiation.

## Materials and Methods

### Animals

Taz‐knockout (*Wwtr1^–/–^*) (Jax, stock 011120) and *Yap^fl/fl^* mice are described 39, 40. *Pax7^Cre^* mice were purchased from The Jackson Laboratory (https://www.jax.org/), Sacramento, California USA (stock 012476). *Pax7^Cre^:Yap^fl/fl^*:*R26‐floxstop‐LacZ* 9 mice were donated by Fernando Camargo. Breeding/experimental procedures were approved by the Ethical Review Process Committee of King's College London, and performed under the Animals (Scientific Procedures) Act 1986.

### Cell Culture

Satellite cells/myofibers were isolated as described previously 41. Briefly, murine extensor digitorum longus (EDL) myofibers were isolated by 0.05% type I collagenase for 2 hours. Myofibers were washed in GlutaMax Dulbecco's modified Eagle's medium (DMEM) (ThermoFisher Scientific (http://corporate.thermofisher.com/en/home.html), Waltham, Massachusetts, USA) and plated on matrigel‐coated plates in growth medium (GlutaMax DMEM, 30% fetal bovine serum [FBS], 1% chick embryo extract [CEE], 10 ng/mL basic fibroblast growth factor, 1% penicillin‐streptomycin (Sigma‐Aldrich (http://www.sigmaaldrich.com/united-kingdom.html), St. Louis, Missouri, USA.) at 37°C with 5% CO_2_. After 72 hours, myofibers were removed, and satellite cells passaged and preplated for 30 minutes to remove fibroblasts, before being plated on matrigel‐coated plates. Differentiation was induced in differentiation medium (GlutaMax DMEM, 2% horse serum, 1% penicillin–streptomycin). Nonadherent cultures: myofibers were cultured in GlutaMax DMEM, 10% horse serum, 0.5% CEE, 1% penicillin–streptomycin.

### Muscle Injury

To recombine via the *loxP* sites flanking *Yap* exons 1 and 2, 200 µg of Tamoxifen/gram body weight (Sigma T5648) was injected intraperitoneally in sunflower oil/5% ethanol for 3 consecutive days, followed by maintenance on a tamoxifen‐containing diet (Tekland). Injury was induced in tibialis anterior (TA) by 30 µL intramuscular injection of 20 µM cardiotoxin (CTX)/saline.

### Retroviral Expression and Small Interfering RNA

Wild‐type (WT) TAZ, TAZ S89A, YAP S127A, or WT YAP was subcloned into a pMSCV‐IRES‐eGFP retroviral expression backbone (Addgene Plasmids 24809, 24815, 17791 and 17790) creating pMSCV‐3xFlag TAZ‐IRES‐eGFP and pMSCV‐3xFlag‐TAZ S89A‐IRES‐eGFP 42. Empty vector was negative control. Retroviruses were packaged in HEK293T cells using standard methods. Medium was changed 1 hour before transfection/transduction. Retroviral suspension diluted 1:4 with polybrene (4 μg/mL) was added for 6 h, before changing medium.

Taz small interfering RNA (siRNA) (Ambion (http://www.ambion.com/), Foster City, California, USA, s97145) and Yap siRNA (Ambion, s202423) were used as per manufacturer's instructions. For plated satellite cells, 25 pmol of siRNA with Lipofectamine RNAiMax (ThermoFisher Scientific) was added to each well for either 6 hours (satellite cells) or 24 hours (C2C12) before medium was changed.

### Real‐Time Quantitative Polymerase Chain Reaction

Total RNA was extracted with RNeasy (Qiagen (https://www.qiagen.com/gb/), Manchester, United Kingdom) and reverse transcribed using QuantiTect reverse transcription (Qiagen) as per manufacturer's instructions. Real‐time quantitative polymerase chain reaction (RT‐qPCR) was performed with Brilliant II SYBR green reagents and a ROX reference dye (Agilent Technologies, (www.genomics.agilent.com), La Jolla, California, USA) using the ViiA7 qPCR system. Primer sequences were Yap (5′‐TGAGCC CAAGTCCCACTC‐3′; R‐5′‐TGTGAGTGTCCCAGGAGAAA‐3′), Taz (5′‐TATCCCAGCCAAATCTCGTG‐3′, R‐5′‐TTCTGCTGGCTCAGGGTAC T‐3′) or as described 43.

### Immunolabeling and EdU Pulsing

Cells/myofibers were fixed with 4% paraformaldehyde (PFA)/phosphate‐buffered saline (PBS) for 10 minutes, permeabilized with 0.5% Triton‐X100/PBS and blocked with 10% goat serum/PBS or 0.035% carrageenan/PBS followed by incubation with antibodies overnight at 4°C 41. Antibodies: anti‐Pax7 (Developmental Studies Hybridoma Bank (DSHB) (http://dshb.biology.uiowa.edu/), Iowa City, Iowa, USA); anti‐myosin heavy chain (MyHC) (MF20, DSHB); anti‐myogenin (F5D, DSHB); anti‐MyoD (clone 5.8A, DakoCytomation, Glostrup, Denmark); anti‐Taz (HPA007415, Sigma); anti‐Yap1 (2F12, Abnova (http://www.abnova.com/), Taipei City, Taiwan); anti‐Tead4 (M01, Abnova).

Cryosections were fixed with 4% PFA/PBS followed by cooled methanol before antigen retrieval in heated citrate buffer 44 and blocking in 10% goat serum/PBS. Antibodies: anti‐MyHC Type I (BA‐D5, DSHB), anti‐MyHC Type IIa (A4.74, DSHB), and anti‐laminin (Sigma, L9393). Fluorochrome‐conjugated secondary antibodies were from ThermoFisher Scientific.

5‐Ethyl‐2′‐deoxyuridine (EdU) (10 µM) was added for 2 hours before fixation and incorporation detected using Click‐iT (ThermoFisher Scientific) according to manufacturer's instructions.

### Western Blotting

Western blotting was performed using Run Blue precast native Page gels (Expedeon (https://www.expedeon.com/) Over, Cambridge, United Kingdom). Protein transfer was performed with the XCell II blot module (ThermoFisher Scientific). Polyvinylidene difluoride (PVDF) membranes were incubated with antibodies overnight/4°C and visualized using fluorochrome‐conjugated secondary antibodies (ThermoFisher Scientific) and digitally imaged.

### Mass Spectrometry

C2C12 cells were grown in DMEM (D5761) with 10% FBS and 4 mM glutamine. Proliferating C2C12 cells were at 50% cell density. Confluent cultures were differentiated for 72 hours in DMEM, 2% horse serum, 4 mM glutamine.

For immunoprecipitation, 80,000 C2C12s were seeded per 10 cm dish. The following day, fresh medium was added 1 hour before addition of 1:5 diluted TAZ or YAP encoding retroviral supernatant. The next day, cells were replated and transduction confirmed by green fluorescent protein (GFP). Cells were washed on ice with PBS, and collected in lysis buffer (150 mM NaCl, 20 mM Tris‐HCl pH 7.5, 1% Triton ×100) with 1 mM sodium orthovanadate, protease inhibitor cocktail (Sigma, p8340), and Phenylmethylsulfonyl fluoride (PMSF) (Sigma‐Aldrich). Lysates were incubated for 1 hour on ice and centrifuged at 14,000 RPM at 4°C for 5 minutes and supernatant incubated at 4°C with anti‐Flag M2 agarose beads (Sigma Aldrich). Beads were washed three times with washing buffer (150 mM NaCl; 20 mM Tris‐HCL pH 7.5). Sample preparation/mass spectrometry were as described 45, with proteomics data deposited to the ProteomeXchange Consortium via the PRIDE 46 partner repository: identifier PXD005751.

### Microarray

Total RNA was isolated from TAZ S89A or YAP S127A transduced myoblasts after 24 or 48 hours using TRIzol (ThermoFisher Scientific) followed by purification and DNase digestion (RNeasy minikits, Qiagen). RNA quantification was performed on a Nanodrop spectrophotometer (ThermoFisher Scientific) and quality tested on an Agilent Tapestation (RIN 7.6–9.8). Generation of sense strand cDNA, second strand synthesis, in vitro transcription cRNA synthesis, single stranded cDNA synthesis and RNA hydrolysis, fragmentation, and labeling were as manufacturer's instructions (GeneChip WT Plus reagent kit, Affymetrix, now part of ThermoFisher Scientific). Hybridization, washing, staining, and scanning of microarrays were carried out on Affymetrix Mouse Gene 2.0 ST microarrays using a GeneChip Fluidics station 450 and GCS3000 scanner (Affymetrix^®^).

Data preprocessing/quality control were performed using Affymetrix^®^ Genechip^®^ Expression Console v1.2. Probe cell intensity data (CEL files) were processed using the RMA16 algorithm (Affymetrix), which fits a linear model at probe level by using background correction, quantile normalization of log2 transformed data, and summarization, for primary QC analysis. Performed in triplicate at Centre for Genome Enabled Biology and Medicine (University of Aberdeen).

Data were analyzed in Partek^®^ Genomics Suite^®^, version 6.6, build 6.15.0730 Copyright; 2014 (Partek Incorporated (http://www.partek.com/), St. Louis, Missouri, USA) using a Mouse Gene 2.0 ST annotation file from build mm10, MoGene‐2.0‐st‐v1.na35.mm10.transcript. Affymetrix CEL files were imported to Partek^®^ Genomics Suite^®^, data processed using RMA normalization with RMA background correction and quantile normalization of log2 transformed data and probeset summarization by median polish. Two‐way analysis of variance (ANOVA) with time point (24 and 48 hours) and transcription factor (Control, TAZ S89A, YAP S127A) and time × transcription interaction to evaluate significantly differentially expressed genes. Fold change in TAZ S89A or YAP S127A compared with control vector as baseline at each time point calculated using geometric mean of samples in each group with significance calculated by Fishers Least significant difference. Fold change ≥1.3 and False Discovery Rate (FDR) of 10% were evaluated. Microarray data available in the ArrayExpress database (www.ebi.ac.uk/arrayexpress), accession number E‐MTAB‐5395.

### Histology

H&E and ATPase staining were described previously 30. Muscle sections were stained with ATPase at pH 4.47 to detect type I, and pH 10.5 to detect type II, myofibers.

### Image Acquisition and Analysis

Images were obtained at room temperature on a Zeiss microscope (Axiovert 200M) equipped with LD A‐plan 20×/0.85 ph1, 10×/0.30ph1 and 40×/.075ph1 objectives or on a Zeiss Exciter laser scanning microscope (LSM) with 40×/1.1 W Corr LD C‐Apochromat objective (Carl Zeiss Ltd (https://www.zeiss.co.uk/), Cambridge, United Kingdom). Images were quantified with image J software (NIH). Neonatal MyHC labeling was quantified with R software. Values are mean ± SEM with a Student's *t* test (paired or unpaired as appropriate) or ANOVA with a post‐hoc Tukey's test for >2 groups.

## Results

### Taz Is Dynamically Regulated during Myogenic Progression

**Figure 1:**
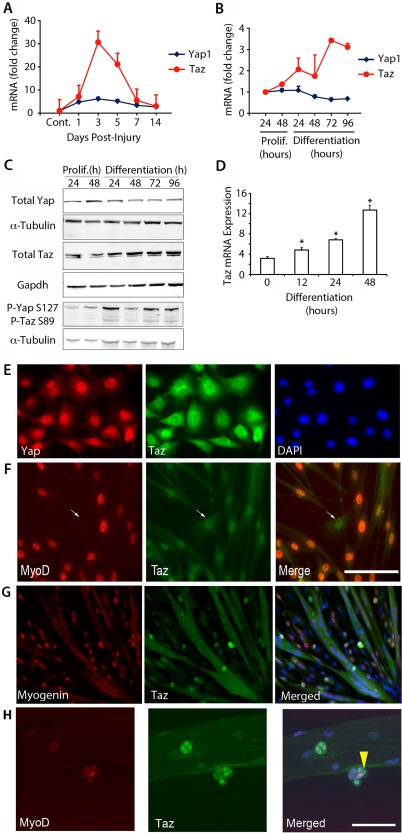
Taz levels increase during skeletal muscle regeneration and myogenic differentiation. **(A):** Yap and Taz mRNA expression in regenerating tibialis anterior (TA) muscle collected at 1, 3, 5, 7, and 14 days post injury. Expression is fold change compared with control undamaged TA (*n* = 3). **(B):** Yap and Taz mRNA levels in C2C12 myoblasts at 24 and 48 hours in proliferation medium, and 24, 48, 72, and 96 hours in differentiation medium, expressed as fold change compared with 24 hours proliferation time‐point (*n* = 3). **(C):** Representative Western blots from biological replicate 4 (*n* = 4) showing total Yap, Taz, and phosphorylated (P) Yap and Taz levels in C2C12 myoblasts at 24 and 48 hours in proliferation medium, and 24, 48, 72, and 96 hours in differentiation medium, with relevant α‐Tubulin or Gapdh loading controls. **(D):** Taz mRNA increased through differentiation in plated satellite cell‐derived myoblasts at 12, 24, and 48 hours. Data are mean ± SEM from three experiments where an asterisk denotes significant difference (*p* < .05) from 0 hour using an unpaired Student's *t* test. **(E):** C2C12 proliferating myoblasts coimmunolabeled for Yap and Taz. **(F):** Plated satellite cell‐derived myoblasts coimmunolabeled with MyoD and Taz after 36 hours ex vivo. **(G):** Plated satellite cell‐derived myotubes coimmunolabeled with myogenin and Taz after 48 hours ex vivo. **(H):** Confocal images of satellite cells coimmunolabeled with MyoD and Taz on isolated fibers cultured ex vivo for 72 hours; arrow points to Taz‐containing nuclei. Scale bar = 100 µm (F, G). Abbreviations: DAPI, 4′,6‐diamidino‐2‐phenylindole; P, Phosphorylation.

To assess *Yap* and *Taz* (*Wwtr1*) expression dynamics during muscle regeneration, we measured mRNA levels by RT‐qPCR in vivo and myogenic progression in vitro. Murine TA muscle was injured by intramuscular injection of CTX and mRNA isolated from regenerating muscle after 1, 3, 5, 7, and 14 days post‐injury (dpi). *Taz* mRNA increased markedly at 3 dpi compared with uninjured control, before dropping, while *Yap* was largely unchanged (Fig. 1A). *Taz* mRNA rose during differentiation of C2C12 myoblasts, while *Yap* mRNA remained relatively constant (Fig. 1B). Western blot analysis for total Yap and Taz protein in C2C12 reflected mRNA dynamics, with Taz levels rising during differentiation, while Yap again remained relatively constant (Fig. 1C). Phosphorylated Yap and Taz levels both showed an increase during myogenic differentiation. *Taz* mRNA also increased during ex vivo differentiation of plated murine satellite cell‐derived myoblasts (Fig. 1D).

Coimmunolabeling C2C12 myoblasts for Yap and Taz revealed Taz localized to the cytoplasm and nucleus, while Yap was nuclear (Fig. 1E), consistent with our previous observations 29. Taz was similarly localized in satellite cells (Fig. 1F), but became cytoplasmic in mature multinucleated myotubes (Fig. 1G). Coimmunolabeling of satellite cells on a myofiber showed that Taz was expressed in satellite cells, as shown by MyoD coexpression (Fig. 1H).

### Taz Promotes Myoblast Proliferation

To compare Taz with Yap function, we either increased Taz activity via retroviral‐mediated expression of human TAZ or constitutively active TAZ S89A, or after decreasing Taz and/or Yap levels using siRNA and performed proliferation and differentiation assays.

**Figure 2:**
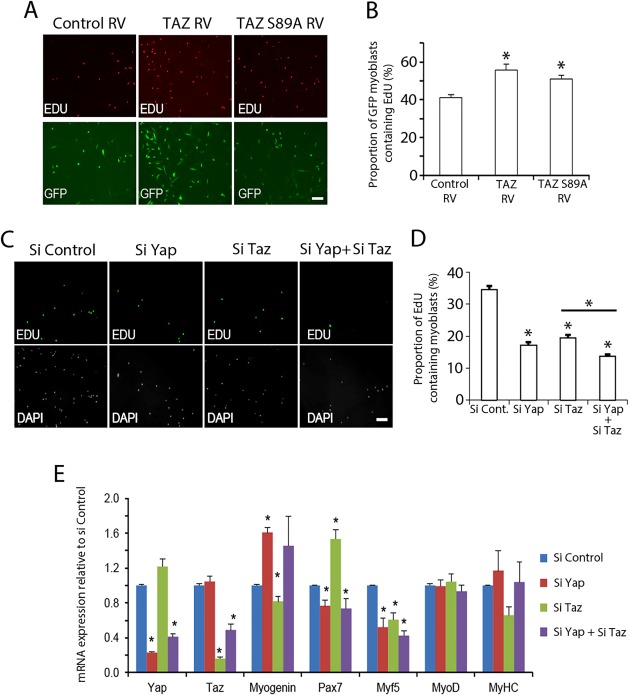
Taz controls satellite cell‐derived myoblast proliferation. **(A):** Representative images of 5‐ethyl‐2′‐deoxyuridine (EdU) incorporation in satellite cell‐derived myoblasts 48 hours following transduction with control retroviral vector (RV), or retroviral constructs encoding wild‐type human TAZ (TAZ RV) or constitutively active TAZ S89A (TAZ S89A RV), where green fluorescent protein (GFP) marks transduced cells. **(B):** Quantification of GFP+ cells with EdU incorporation shows that TAZ or TAZ S89A significantly increases EdU incorporation, and so proliferation rate (*n* = 5 mice). **(C):** Representative images of EdU incorporation in satellite cell‐derived myoblasts following *Yap* and/or *Taz* small interfering RNA (Si)‐mediated knockdown using siControl, siYap, siTaz, or siYap + siTaz transfection. **(D):** Quantification of EdU incorporation shows that knockdown of Yap, Taz, or both Yap and Taz simultaneously reduces the proliferation rate (*n* = 5 mice). **(E):** Relative gene expression measured by RT‐qPCR in satellite cell‐derived myoblasts compared with si Control under proliferation conditions following siControl, siYap, siTaz, or siYap + siTaz knockdown, normalized to Gapdh (*n* = 5 mice except for myosin heavy chain/MyoD, where *n* = 3). Data presented as mean ± SEM, where an asterisk denotes significant difference from control RV/Si control (*p* < .05) using Student's *t* test. Scale bar = 100 µm. Abbreviations: DAPI, 4′,6‐diamidino‐2‐phenylindole; EdU, 5‐ethyl‐2’‐deoxyuridine; GFP, green fluorescent protein; RV, retroviral vector; Si, small interfering RNA.

The retroviral backbone contains an *IRES‐eGFP* that allows transduced cells to be identified by eGFP expression. Immunolabeling revealed that TAZ or TAZ S89A significantly increased the proportion of eGFP+ ve cells that had incorporated EdU, indicating that TAZ increases proliferation rate (Fig. 2A, 2B). siRNA‐knockdown of *Taz*, *Yap*, or both *Taz/Yap* simultaneoulsy, however, significantly reduced EdU incorporation (Fig. 2C, 2D). Thus, at this stage, TAZ promotes proliferation of myoblasts, as we reported previously for YAP 28, 29.

RT‐qPCR showed that siRNA‐mediated knockdown of *Taz* suppressed *Myf5* and *myogenin* expression, but enhanced *Pax7*. Conversely, knockdown of Yap increased *myogenin* expression, but reduced *Pax7* and *Myf5*. Simultaneous knockdown of both *Taz* and *Yap* rescued expression of *myogenin* (Fig. 2E).

### Taz and Yap Play Opposing Roles during Myogenic Differentiation

**Figure 3:**
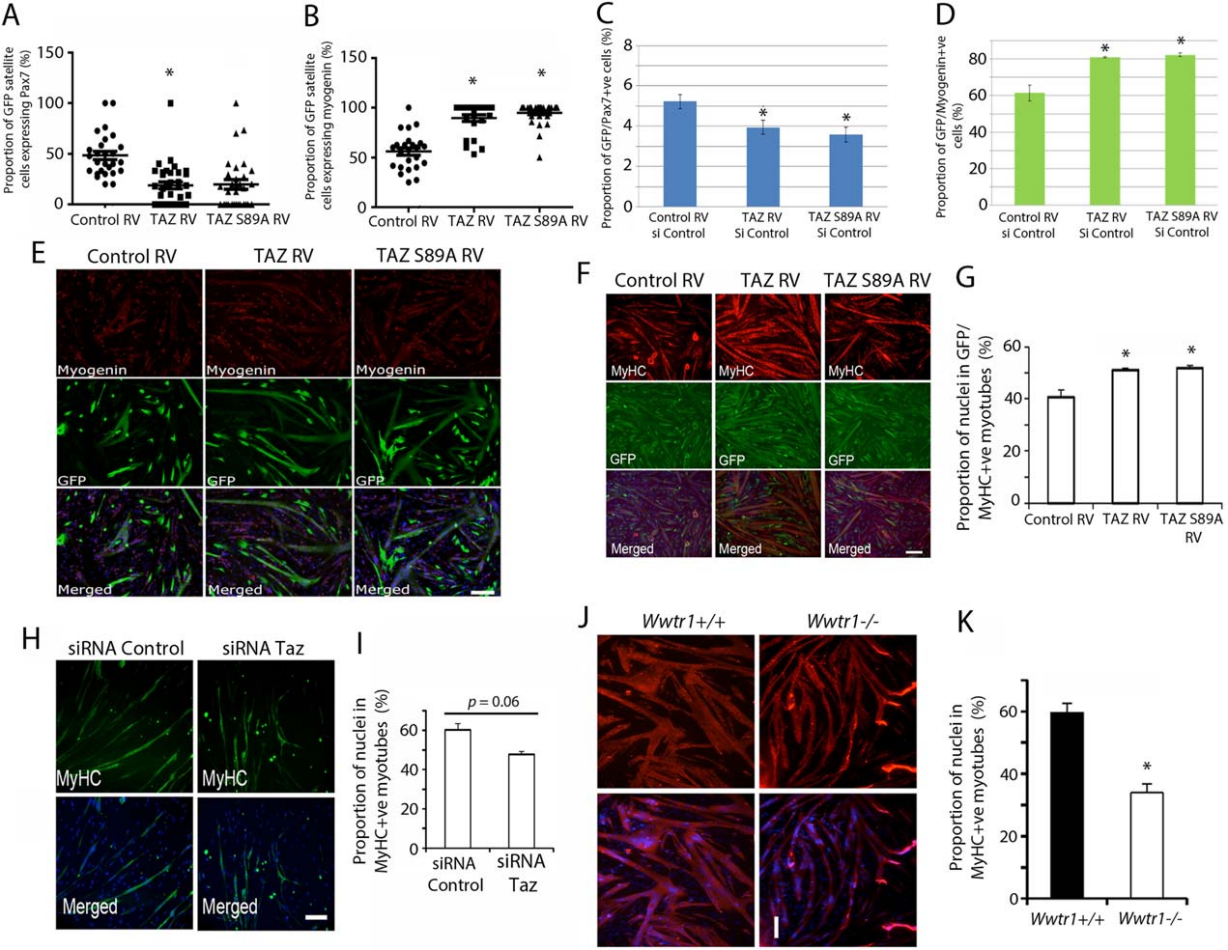
Taz regulates fusion of satellite cell‐derived myoblasts. **(A, B):** Satellite cells on isolated myofibers were transduced with retroviral vectors (RV) encoding wild‐type (WT) human TAZ (TAZ RV), constitutively active TAZ S89A (TAZ S89A RV) or Control RV, and cultured ex vivo for 72 hours, before being coimmunolabeled for eGFP and Pax7, or eGFP and Myogenin. Quantification of the proportion of (A) green fluorescent protein (GFP)/Pax7 and (B) GFP/myogenin satellite cells in the ex vivo cultures after 72 hours (*n* = 3 mice). **(C, D):** Quantification of the proportion of expanded plated satellite cell‐derived myoblasts transduced with control RV, TAZ RV, or TAZ S89A RV and incubated in differentiation medium for 24 hours before being coimmunolabeled as myocytes for eGFP/Pax7 or eGFP/myogenin, illustrated in **(E)** (*n* = 4 mice). **(F):** Representative images of expanded plated satellite cell‐derived myoblasts transduced with TAZ RV, TAZ S89A RV, or Control RV and incubated in differentiation medium before being coimmunolabeled for eGFP/myosin heavy chain (MyHC). **(G):** Quantification of the proportion of nuclei within GFP/MyHC myotubes shows that TAZ or TAZ S89A increases myogenic fusion (*n* = 5 mice). **(H):** Representative images of satellite cell‐derived myoblasts transfected with small interfering RNA (siRNA) control or siRNA against Taz (SiRNA Taz) and immunolabeled for MyHC after 24 hours. **(I):** Quantification shows a trend toward reduced fusion (*p* = .06) with siRNA Taz (*n* = 3 mice). **(J):** Representative images of myotubes formed from satellite cell‐derived myoblasts isolated from WT (*Wwtr1^+/+^*) or Taz‐knockout (*Wwtr1* ^–/–^) mice after 24 hours in differentiation medium. **(K):** Quantification reveals less fusion in the Taz‐knockout (*Wwtr1* ^–/–^) mice compared with control WT (*n* = 4 per genotype). Data are mean ± SEM, where an asterisk denotes significant difference (*p* < .05) using a Student's *t* test from Control RV/siRNA control/*Wwtr1^+/+^* as appropriate. Scale bars = 100µm. Abbreviations: GFP, green fluorescent protein; MyHC, myosin heavy chain; RV, retroviral vector; siRNA, small interfering RNA; WT, wildtype.

We determined the effect of retroviral‐mediated constitutive expression of TAZ or TAZ S89A on early myogenic differentiation in satellite cells cultured in their niche for 72 hours on an isolated myofiber, by coimmunolabeling for eGFP/Pax7 to mark undifferentiated/self‐renewing cells (Fig. 3A) or eGFP/myogenin to mark myoblasts entering differentiation (Fig. 3B). TAZ significantly decreased eGFP‐containing self‐renewing Pax7 + ve cells (Fig. 3A), while TAZ or TAZ S89A significantly increased eGFP‐containing differentiating myogenin+ve myoblasts (Fig. 3B), compared with control. Thus, TAZ promotes entry into differentiation, at the expense of satellite cell self‐renewal.

To investigate effects of TAZ or TAZ S89A throughout myogenic differentiation, we used expanded, plated satellite cell‐derived myoblasts, which were first transduced and then switched to differentiation medium 24 hours later. Self‐renewal and differentiation were again assessed by coimmunofluorescence for eGFP/Pax7 and eGFP/myogenin. Under differentiation stimulus, TAZ or TAZ S89A decreased the proportion of myocytes containing Pax7, while enhancing the proportion expressing myogenin (Fig. 3C, 3D). At this early stage of differentiation, myotubes were also more prominent in myoblast cultures transduced with TAZ or TAZ S89A, than control retrovirus, suggesting a time‐dependent switch of TAZ function from pro‐proliferation to pro‐differentiation (Fig. 3E). At later stages of differentiation and fusion into multinucleated myotubes, constitutive expression of TAZ or TAZ S89A also increased incorporation of nuclei into myotubes (fusion index‐Fig. 3F, 3G). siRNA‐mediated Taz knockdown in expanded plated satellite cell‐derived myoblasts did not significantly reduce (though *p* = .06) the fusion index to below control levels (Fig. 3H, 3I).

### Taz‐knockout (*Wwtr1* ^–/–^) Myoblasts Differentiate Less Ex Vivo

Skeletal muscles of Taz‐knockout (*Wwtr1* ^–/–^) mice have not been well‐characterized 40. Consistent with the trend in siRNA data (Fig. 3H, 3I), satellite cells isolated and expanded from Taz‐knockout (*Wwtr1* ^–/–^) mice fused less into post‐mitotic myotubes compared with WT cells (Fig. 3J, 3K).

Thus, Taz promotes proliferation of myoblasts, but at later stages of myogenesis, Taz switches to enhance differentiation. In contrast, as we reported previously, Yap continues to promote proliferation while inhibiting differentiation at this stage 28, 29.

### Taz‐knockout (*Wwtr1* ^–/–^) Mice Have Fewer Myofibers in Soleus

**Figure 4:**
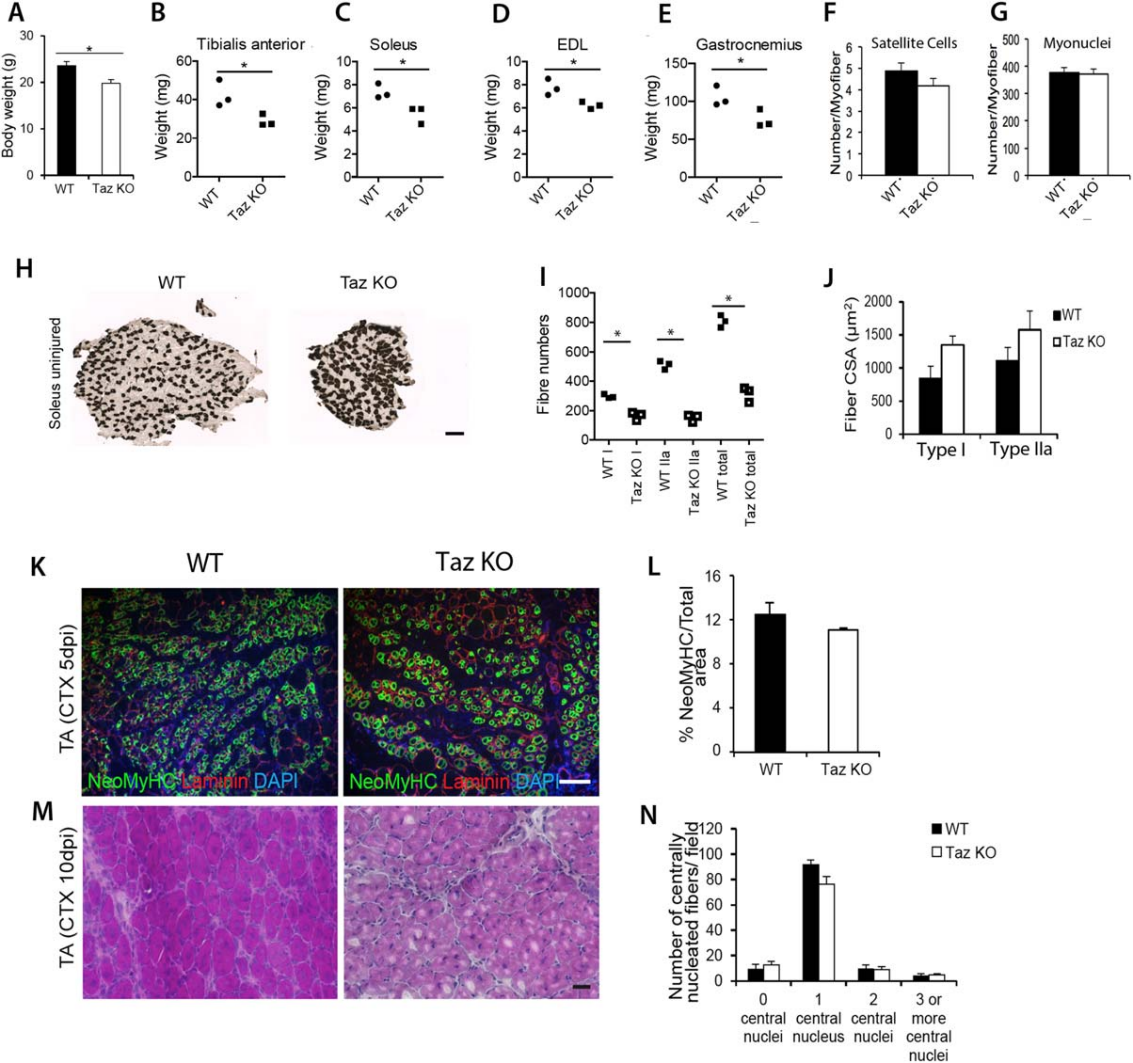
Characterization of skeletal muscle and muscle regeneration in Taz‐knockout (*Wwtr1* ^–/–^) mice. **(A):** Body weight of 6‐week‐old Taz‐knockout (KO) (*Wwtr1* ^–/–^) mice (*n* = 7) compared with wild‐type (WT) *Wwtr1^+/+^* control (*n* = 9). **(B–E):** Quantification of muscle weight of tibialis anterior (TA), Soleus, extensor digitorum longus (EDL), and Gastrocnemius from WT and Taz KO mice (*n* = 3 mice). **(F):** Satellite cell and **(G):** myonuclei number per myofiber isolated from the EDL of WT or Taz KO mice (*n* = 4 mice of each genotype). **(H):** Representative images of soleus from 6‐week‐old WT and Taz KO after ATPase histochemical staining at pH 4.47 to show slow type I (dark) and fast type IIa (light/no stain) muscle fiber types. **(I):** Quantification showing less total myofibers in the soleus of Taz KO mice, with numbers of both type I and type IIa reduced (*n* = 3 mice of each genotype). **(J):** Quantification of cross section area of type I and type IIa reveals that myofibers from Taz KO are unchanged compared with controls (*n* = 3 mice per genotype). **(K):** Representative images and **(L):** quantification of the proportion of the neonatal myosin heavy chain (Neo MyHC) area of regenerating TA myofibers coimmunolabeled for NeoMyHC/laminin at 5 days post injury (dpi) with CTX (*n* = 4 mice of each genoptype). **(M):** Representative images of H&E staining at 10 dpi of TA from Taz KO and WT mice. **(N):** Quantification of the number of myofibers with a central nucleus per field of 10 dpi TA (*n* = 3 mice per genotype). Data are mean ± SEM where an asterisk denotes significant difference (*p* < .05) between WT and Taz KO using a Student's *t* test. Scale bar = 100 µm. Abbreviations: CSA, cross section area; CTX, cardiotoxin; EDL, extensor digitorum longus; KO, knockout; NeoMyHC, neonatal myosin heavy chain; TA, tibialis anterior; WT, wild type.

In accordance with the role of the Hippo pathway in body/organ size regulation, 6‐week‐old Taz‐knockout (*Wwtr1* ^–/–^) mice were lighter than WT mice (Fig. 4A) as were their TA, soleus, EDL, and gastrocnemius muscles (Fig. 4B–4E). However, the number of satellite cells and myonuclei per EDL myofiber was unchanged between Taz‐knockout (*Wwtr1* ^–/–^) and WT mice (Fig. 4F, 4G). Myofiber type composition in soleus was analyzed using ATPase activity at pH 4.47 to detect slow type I, or pH 10.5 to detect fast type IIa, myofibers. Soleus contained fewer muscle fibers in total, with both numbers of type I and type IIa myofibers significantly reduced in Taz‐knockout (*Wwtr1* ^–/–^) compared with WT (Fig. 4H, 4I), but the proportion of slow type I myofibers was significantly increased, while the proportion of type IIa were significantly decreased, compared with control WT. Myofiber cross‐sectional area was unchanged though (Fig. 4J).

Next, we investigated muscle regeneration in the Taz‐knockout (*Wwtr1* ^–/–^) mice using intra‐muscular injection of CTX into TA. Cryosections of 5 dpi regenerating TA were immunolabeled for neonatal MyHC to identify regenerating myofibers, but Taz‐knockout (*Wwtr1* ^–/–^) did not exhibit a reduction compared with controls (Fig. 4K, 4L). At 10 dpi, the number of myofibers with centrally located nuclei in TA of Taz‐knockout (*Wwtr1* ^–/–^) mice was also unchanged (Fig. 4M, 4N).

### Inactivation of Yap in Satellite Cells Impairs Muscle Regeneration

**Figure 5:**
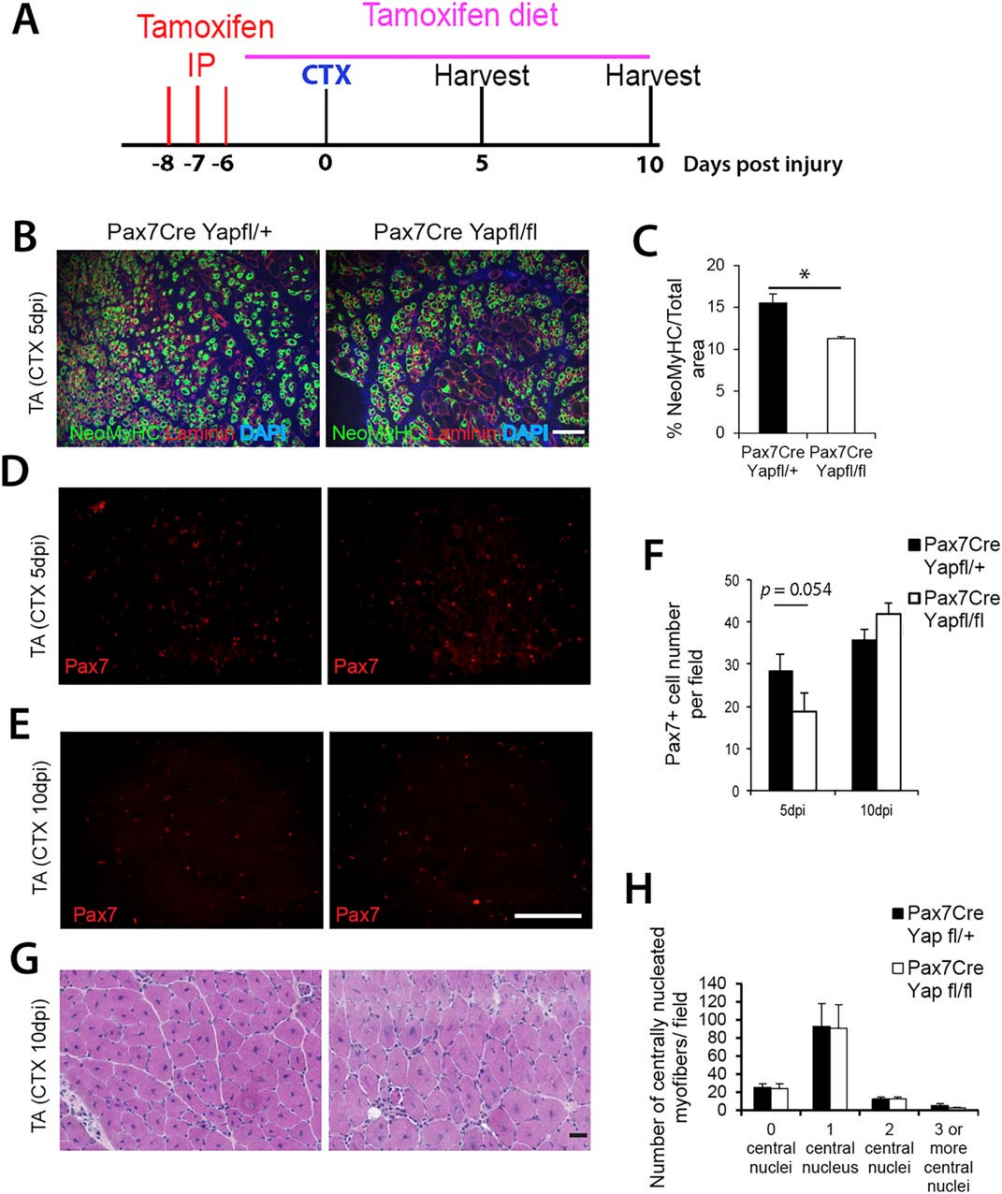
Yap conditional knockout in satellite cells affects muscle regeneration. **(A):** Time course for analysis of Yap conditional knockout and control mice after tamoxifen administration via intraperitoneal injection and food, and cardiotoxin (CTX)‐induced injury of the tibialis anterior (TA) muscle. **(B, C):** Representative images and quantification of TA cryosections 5 dpi after CTX injury of *Pax7^CreERT/+^:Yap^fl^°^x/fl^°^x^:Rosa26^Laz^* (Pax7Cre Yapfl/fl) and control *Pax7^CreERT/+^:Yap ^fl^°^x/+^:Rosa26^Laz^* (Pax7Cre Yapfl/+) mice, coimmunolabeled for neonatal myosin heavy chain (NeoMyHC)/laminin to identify regenerating myofibers (*n* = 4 mice per genotype). **(D–F):** Representative images and quantification of cryosections of TA muscles after 5 or 10 dpi, immunolabeled for Pax7 to label satellite cells (5 dpi: *n* = 4 per genotype; 10 dpi: *n* = 4 Pax7Cre Yapfl/fl and *n* = 3 Pax7Cre Yapfl/+). **(G):** Representative images of H&E staining and **(H)** quantification of centrally nucleated myofiber number per field of 10 dpi TA. Data are mean ± SEM, where an asterisk denotes significant difference (*p* < .05) between Pax7Cre Yapfl/+ and Pax7Cre Yapfl/fl using a Student's *t* test. Scale bar = 100 µm. Abbreviations: CTX, cardiotoxin; DAPI, 4′,6‐diamidino‐2‐phenylindole; IP, intraperitoneal; NeoMyHC, neonatal myosin heavy chain; TA, tibialis anterior.

Administration of Tamoxifen to *Pax7^Cre‐ERT2/+^:Yap^fl^°^x/fl^°^x^:Rosa26^Lacz^* mice causes *Pax7*‐driven Cre‐ERT2‐mediated deletion of *Yap1* exons 1 and 2 in satellite cells, with simultaneous induction of *lacZ* expression. *Pax7^Cre‐ERT2/+^:Yap^fl^°^x/fl^°^x^:Rosa26^Lacz^* and age‐matched control *Pax7^Cre‐ERT2/+^:Yap^fl^°^x/+^:Rosa26^Lacz^* mice were given three intraperitoneal injections of Tamoxifen, and then maintained on tamoxifen‐containing food. TA muscles were injured by intramuscular injection of CTX, and regenerating muscles cryosectioned at 5 and 10 dpi (Fig. 5A).

Regenerating TA muscles at 5 dpi from Tamoxifen‐treated *Pax7^Cre‐ERT2/+^:Yap^fl^°^x/fl^°^x^:Rosa26^Lacz^* mice coimmunolabeled for neonatal MyHC and laminin had an approximate 30% smaller area containing neonatal MyHC than control *Pax7^Cre‐ERT2/+^:Yap^fl^°^x/+^:Rosa26^Lacz^* (Fig. 5B, 5C). Immunolabeling for Pax7 revealed that there was a nonsignificant (*p* = .054) trend to reduced numbers of satellite cells in Tamoxifen‐treated 5 dpi regenerating *Pax7^Cre‐ERT2/+^:Yap^fl^°^x/fl^°^x^:Rosa26^Lacz^* muscle (Fig. 5D, 5F).

By 10 dpi, Pax7 immunolabeling revealed that satellite cell number in tamoxifen‐treated *Pax7^Cre‐ERT2/+^:Yap^fl^°^x/fl^°^x^:Rosa26^Lacz^* mice was as in control *Pax7^Cre‐ERT2/+^:Yap^fl^°^x/+^:Rosa26^Lacz^* mice (Fig. 5E, 5F). The number of myofibers with centrally located myonuclei, a hallmark of regenerated muscle, was also unchanged between *Pax7^Cre‐ERT2/+^:Yap^fl^°^x/fl^°^x^:Rosa26^Lacz^* regenerating TA muscles and control (Fig. 5G, 5H). Thus, lack of Yap function in satellite cells slows, but does not prevent, muscle regeneration.

### YAP and TAZ Regulate the Hippo Negative Feedback Loop, Myogenic Regulators, and Wnt Signaling

To compare the effects of TAZ and YAP on gene expression in the skeletal muscle lineage, we expressed TAZ S89A or YAP1 S127A in proliferating murine satellite cell‐derived myoblasts for 24 and 48 hours and analyzed gene expression by microarray (Supporting Information Data 1), comparing our observations with those obtained by induction of YAP1 S127A in myoblasts derived from a transgenic mouse model 29 and from rhabdomyosarcomas driven by YAP1 S127A 25.

**Figure 6:**
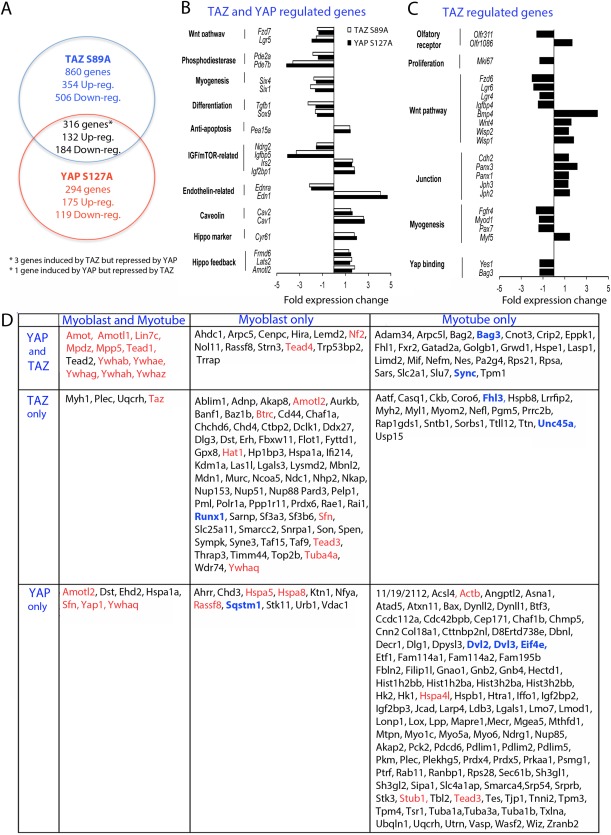
Genes regulated by TAZ S89A or YAP S127A and proteins bound by TAZ and/or YAP. **(A):** Venn diagram displaying the number of genes modulated by TAZ S89A and/or YAP S127A versus control retrovirus (up‐reg: upregulated; down‐reg: downregulated) at either 24 or 48 hours. **(B):** Expression fold change for genes that were regulated by both TAZ S89A and YAP S127A in transduced satellite cell‐derived myoblasts versus control retrovirus at either 24 or 48 hours. **(C):** Expression fold change for genes that were regulated only by TAZ S89A at either 24 or 48 hours. **(D):** All TAZ and/or YAP interacting proteins identified by mass spectrometry in C2C12 myoblasts and myotubes. Those previously identified are in red 53 and novel proteins in black, with those of particular interest in blue. To minimize false positives, selected cut‐off values were a ratio of ≥2 compared with empty vector and *p* < .05 Student's *t* test between each dataset and control vector. Abbreviations: IGF, insulin‐like growth factor; mTOR, mechanistic target of rapamycin.

Combination of 24 and 48 hours time points revealed that TAZ S89A significantly changed expression of 860 genes, while YAP S127A altered expression of 294 genes, with an additional 316 genes regulated by both TAZ S89A and YAP S127A (Fig. 6A; Supporting Information Data 1). Satellite cells were already actively proliferating as cultured in high serum medium, and serum is a potent YAP activator 47, likely explaining why neither TAZ S89A nor YAP1 S127A significantly affected most mitotic genes, in contrast to earlier observations 25, 29.

Increased TAZ/YAP‐Tead activity was indicated by both TAZ S89A and YAP S127A inducing the Hippo negative feedback loop genes *Amotl2, Frmd6* (Willin), and *Lats2,* which was presumably to limit Taz/Yap activity. TAZ S89A and YAP S127A also regulate markers/regulators of the muscle lineage: *Caveolin1* (Cav1) was upregulated, while *Six1* and *Six4* were downregulated. In addition, TAZ and YAP regulated multiple members of the mTOR/insulin‐like growth factor (IGF) pathway, including *Igfbp5*, as well as *Ndrg2* and *Irs2,* which regulate mTOR via Akt (Fig. 6B).

Wnts are TAZ/YAP regulators, and TAZ/YAP regulation of Wnt and Wnt‐related proteins can inhibit Wnt signaling 48. TAZ S89A and YAP S127A altered expression of key Wnt pathway regulators, reducing expression of Wnt‐receptor proteins *Fzd7* and *Lgr5*, while TAZ S89A alone suppressed expression of *Fzd6*, *Lgr4*, and *Lgr6,* suggesting that TAZ/YAP desensitize myoblasts to ligands that bind these receptors. Moreover, TAZ S89A increased expression of *Wnt4*, and altered expression of other secreted Wnt‐associated proteins and genes encoding the structurally related proteins *Wisp1* and *Wisp2* 49. Additionally, both TAZ S89A and YAP S127A decreased *Sox9* expression (Fig. 6B, 6C).

### TAZ Regulates a Unique Set of Genes

Although the microarray was performed on proliferating myoblasts, genes regulated by TAZ only might explain the functional switch from pro‐proliferation to pro‐differentiation (Fig. 6C). Both *Pax7* and *MyoD* were downregulated by TAZ S89A while *Myf5* was upregulated, consistent with Taz siRNA knockdown (Fig. 2E). Upregulated cell junction proteins could contribute to enhanced fusion capability in TAZ‐overexpressing myoblasts, since *N*‐Cadherin is required for myoblast fusion 50. TAZ overexpression also altered expression of olfactory receptors *Olfr311* and *Olfr1086* (Fig. 6C), a family of genes with members engaged in controlling satellite cell self‐renewal 51. *Mki67* (protein Ki67) is involved in proliferation, but was downregulated by TAZ in myoblasts (Fig. 6C), again highlighting the switch from promoting proliferation toward cell cycle withdrawal/differentiation.

### YAP and TAZ Bind Both Common and Unique Proteins

Apart from Tead1‐4, Yap and Taz also bind a plethora of upstream signaling proteins and downstream transcriptional regulators 52. To identify binding partners in a non‐biased way, we expressed flag‐tagged WT human YAP or TAZ in proliferating murine C2C12 myoblasts and mature myotubes. Flag‐immunoprecipitated YAP or TAZ complexes were identified using mass‐spectrometry (Fig. 6D; Supporting Information Data 2).

Our TAZ and YAP binding proteins were first validated against previously characterized binding partners in human embryonic kidney 293T cells 53. Overlap included angiomotins, multiple 14‐3‐3 proteins, Tead1, Tead3, and Tead4 (red: Fig. 6D). However, we also identified many novel TAZ and/or YAP interacting proteins in myogenic cells (Fig. 6D). TAZ bound many more proteins in myoblasts than YAP, or that were common to both YAP and TAZ. In myotubes, however, YAP bound many more proteins than TAZ or both TAZ and YAP, with only YAP binding Wnt regulators Dvl2 and Dvl3. Of 26 TAZ and YAP binding proteins in mature myotubes (Fig. 6D), 10 are linked to actin cytoskeleton and the Chaperone assisted selective autophagy (CASA) complex, including the WW‐domain containing Bag3. We also identified 20 proteins bound specifically by TAZ during differentiation, the majority associated with the actin cytoskeleton and myogenesis including Unc45a and Fhl3 54, 55. MyoD was not among YAP and/or TAZ binding partners, even at less stringent cut‐offs.

### TAZ Operates through Tead4 to Control Myogenic Differentiation

**Figure 7:**
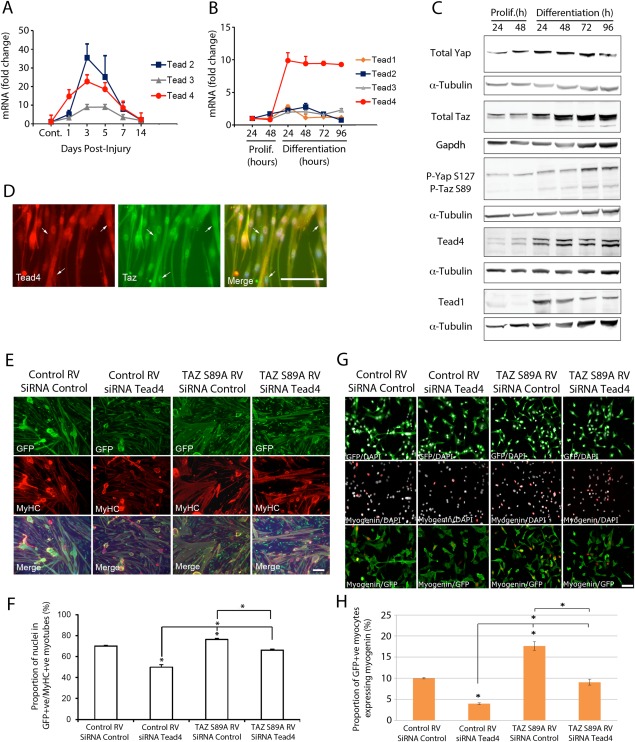
TAZ operates through Tead4 to control myogenic differentiation. **(A):** Tead2–4 mRNA expression in regenerating tibialis anterior (TA) muscle collected at 1, 3, 5, 7, and 14 days post injury with cardiotoxin. Expression is presented as fold change compared with control undamaged TA (*n* = 3). **(B):** Tead1–4 mRNA levels in C2C12 myoblasts at 24 and 48 hours in proliferation medium, and 24, 48, 72, and 96 hours in differentiation medium, expressed as fold change compared with 24 hours proliferation time‐point (*n* = 3). **(C):** Representative Western blots of C2C12 myoblast lysate collected at 24 and 48 hours in proliferation medium and 24, 48, 72, and 96 hours in differentiation medium and immunoblotted for Yap, Taz, Tead1 (biological replicate 3), phosphorylated (P) Yap and Taz (biological replicate 2) and Tead4 (biological replicate 1) (*n* = 4), with relevant α‐Tubulin or Gapdh loading controls. **(D):** Representative images of plated satellite cell‐derived myoblasts coimmunolabeled with Tead4 and Taz cultured under differentiation conditions for 24 hours. **(E):** Representative images of coimmunolabeling of myotubes formed from satellite cell‐derived myoblasts following TAZ S89A RV or Control RV transduction and/or small interfering RNA (siRNA) knockdown of Tead4 or siRNA Control. Green fluorescent protein (GFP) labels transduced cells and myosin heavy chain (MyHC) identifies myotubes. **(F):** Comparison of the proportion of nuclei in GFP+ve/MyHC+ve myotubes under each condition shows that TAZ S89A requires Tead4 to enhance differentiation (*n* = 3 mice). **(G):** Representative images of coimmunolabeling of myocytes formed from satellite cell‐derived myoblasts following TAZ S89A RV or Control RV transduction and/or siRNA knockdown of Tead4 or siRNA Control, and 24 hours in differentiation medium. GFP labels transduced cells and Myogenin identifies myocytes. **(H):** Comparison of the proportion of GFP+ve/Myogenin+ve myocytes under each condition shows that TAZ‐induced myogenic differentiation operates through Tead4 (*n* = 3 mice). Data are mean ± SEM from three mice, where an asterisk denotes significant difference (*p* < .05) from Control RV/siRNA Control, or as indicated by bars, using a paired Student's *t* test. Scale bar = 100 µm. Abbreviations: DAPI, 4′,6‐diamidino‐2‐phenylindole; GFP, green fluorescent protein; MyHC, myosin heavy chain; P, Phosphorylated; RV, retroviral vector; siRNA, small interfering RNA; TA, tibialis anterior.

We next assayed Tead 1–4 expression during myogenesis. TA muscle was injured using CTX and mRNA isolated from regenerating muscle after 1, 3, 5, 7, and 14 dpi. RT‐qPCR showed that *Tead2* and *Tead4* exhibited similar expression profiles, peaking around day 3, before falling to levels seen in undamaged muscle (Fig. 7A). *Tead1‐3* mRNA was at a constant level in proliferating and differentiating C2 myoblasts, but *Tead4* expression increased robustly at the onset of myogenic differentiation, being maintained at elevated levels through fusion (Fig. 7B). Western blot revealed that Tead1 and Tead4 protein levels increased upon entry into C2C12 myoblast differentiation and were maintained (Fig. 7C).

As Tead4 is critical for myoblast fusion due to inducing myogenin by binding MCAT elements in its promoter 56, we further examined its function. Coimmunolabeling of Tead4 and Taz showed both proteins present in the nucleus of differentiating myoblasts, at a time when Tead4 levels rise markedly (Fig. 7D). Thus, the TAZ‐Tead4 interaction identified in proliferating myoblasts (but not in mature myotubes) (Fig. 6D) could be maintained during induction and the early phases of myogenic differentiation, not assayed by proteomics.

We used retroviral‐mediated expression to enhance TAZ activity and/or siRNA to reduce Tead4 levels in satellite cell‐derived myoblasts during myogenic differentiation, and assayed fusion index after coimmunolabeling for eGFP/MyHC. TAZ S89A with control siRNA augmented myoblast fusion (Fig. 7E, 7F), but TAZ S89A with concomitant Tead4 knockdown reversed this enhanced fusion index (Fig. 7E, 7F). Knockdown of Tead4 alone resulted in shorter and thinner myotubes, reflected in a reduced fusion index, but expression of TAZ S89A did allow recover of the fusion index back to control levels (Fig. 7E, 7F).

To investigate the role of TAZ/Tead4 in entry into myogenic differentiation versus fusion, we also transduced satellite cell‐derived myoblasts with TAZ S89A and/or transfected with Tead4 siRNA and differentiated cells as myocytes. After 24 hours in differentiation medium, coimmunolabeling revealed that TAZ S89A increased the proportion of eGFP+ve cells containing myogenin, compared with controls. Knockdown of Tead4 alone significantly reduced myogenin expression (Fig. 7G, 7H). Simultaneous expression of TAZ S89A and Tead4 siRNA knockdown though, brought the proportion of GFP‐expressing myoblasts containing myogenin back to control levels (Fig. 7G, 7H).

## Discussion

Yap, Taz, and Tead1‐4 constitute a dynamic system in satellite cells and the muscle lineage, with mRNA and protein levels of some changing extensively during muscle regeneration and myogenic differentiation in vitro. Three are of note: Yap 29 and Taz only become detectable during satellite cell activation, suggesting that Yap and Taz only operate during myogenesis; mRNA/protein levels of some Teads also change, suggesting that Teads are not just static targets of Yap/Taz; Taz and Tead4 increase dramatically during myogenic differentiation, while Yap expression remains fairly constant. During regeneration, Taz and Tead4 levels peak around 3–5 dpi, as myogenic differentiation is underway 57, 58, 59. Our observations are consistent with Taz and Tead4 as pro‐differentiation factors 28, 29, 37, 56.

Manipulation of Yap and Taz activity/levels via retroviral‐mediated expression of human WT TAZ or TAZ S89A, or knockdown of Taz, revealed that TAZ promotes proliferation. YAP 28, 29 and TAZ synergistically promote proliferation in many cell types 60, with a recent ChIP‐Seq study in breast cancer cells showing that YAP and TAZ frequently operate through the same enhancer elements 24. Later, in myogenesis, however, TAZ switches to promote differentiation, in accordance with an earlier reports in immortalized murine myoblasts 37, 38, unlike YAP, which inhibits differentiation 28, 29. TAZ actually augments the myogenic differentiation program under differentiation stimuli, as shown by the precocious expression of myogenin in many myocytes. Thus, the greater fusion index with TAZ overexpression is not merely due to having more myoblasts available, due to the pro‐proliferation effects of TAZ. Consistent with its later pro‐differentiation role, constitutive TAZ expression also reduced self‐renewal of satellite cells, while Taz‐knockout (*Wwtr1* ^–/–^) satellite cells exhibited reduced fusion ex vivo.

Taz‐knockout (*Wwtr1* ^–/–^) mice have functional and growth defects in kidneys and other organs, consistent with Taz as a regulator of organ growth and transcriptional regulator of genes involved in heart, lung, and bone growth 21, 61, 62. Taz‐knockout (*Wwtr1* ^–/–^) mice were generally lighter, with TA, soleus, EDL, and gastrocnemius muscle mass all reduced, but with unchanged numbers of satellite cells and myonuclei per myofiber. However, absence of Taz reduced the total number of myofibers in soleus, with both fewer slow type I and fast Type IIa muscle fibers present, although there was a greater proportion of slow type I myofibers.

Lack of an overt muscle regeneration phenotype in Taz‐knockout (*Wwtr1* ^–/–^) mice could be due to compensation by Yap in promoting satellite cell proliferation, as Yap or Taz knockdown did not affect expression of the other gene 63. In comparison, genetic deletion of Yap specifically in satellite cells caused delayed regeneration, so Taz is unable to compensate for Yap in early regeneration. This regeneration defect in tamoxifen‐treated *Pax7^Cre‐ERT2/+^:Yap^fl^°^x/fl^°^x^:Rosa26^Lacz^* mice was transient although, maybe due to Taz promoting myogenic differentiation later in regeneration. Therefore, skeletal muscle joins other systems where Yap is required for regeneration, for example, where defects in crypt regeneration are observed when Yap is inactivated in epithelium of the small intestine, and in the skin 64, 65, 66.

We also examined gene expression in myoblasts in response to TAZ S89A or YAP S127A. As expected, YAP and TAZ increased expression of Hippo marker gene *Cyr61* and Hippo negative‐feedback loop genes *Lats2, Frmd6* (Willin), and *Amotl2*: a typical response to increased Taz and Yap activity 29. Intriguingly, TAZ also downregulates *Bag3*, a WW domain‐containing member of the CASA mechanosensitive Z‐disc linked complex 67 that binds to Yap and Taz, and *Yes1*, the earliest reported binding partner of Yap 68. Bag3 and Yes1 might represent additional members of the Hippo negative feedback loop in myoblasts. Several TAZ‐ and YAP‐regulated genes have been linked to myogenesis. For example, TAZ and YAP reduced expression of *Six1* and *Six4*, two factors controling migration of muscle precursor cells 69 with a role in muscle regeneration 70. TAZ and YAP also regulated many IGF/mTOR‐related genes (*Ndrg2, Igfbp5, Irs2*, and *Igf2bp1*), confirming crosstalk between Hippo and mTOR signaling 2, 3.

TAZ also regulates a unique cohort of genes, including *Pax7,* whose expression was reduced by TAZ S89A, consistent with the switch toward differentiation. TAZ also modulates multiple olfactory receptor genes, whose levels vary between *mdx* and control mice 51, and a family of genes with members that can affect muscle regeneration 71.

TAZ and YAP bind proteins in murine myoblasts/myotubes that have been identified as binding partners in human 293T cells 53. These generic YAP and TAZ binding proteins include the angiomotins (Amot and Amotl1), Tead1 and Tead4 and several 14‐3‐3 proteins (Ywhae, Ywhag, Ywhaz). Additionally, we identified many novel TAZ and/or YAP binding partners in the skeletal muscle lineage, including in myotubes, Bag3 72 and syncoilin (Sync), an intermediate filament protein linked to the dystrophin‐associated protein complex 73. TAZ‐specific binding partners that could potentially explain pro‐differentiation effects include Fhl3, which binds MyoD to inhibit differentiation 55 and Unc45a, which increases muscle cell proliferation and fusion, and when inhibited, blocks muscle cell fusion 54. Runx1 bound to TAZ in myoblasts and downregulation of Runx1 leads to cell cycle exit and differentiation 54. Taz has been reported to bind MyoD using immunoprecipitation assays 37, but while we observed MyoD and Taz in the same nuclei, we found no significant direct interaction using mass spectrometry.

Our combined gene expression and binding partner analyses in myoblasts reveals that YAP or TAZ does not bind members of the Wnt destruction complex 8. YAP bound the disheveled proteins Dvl2 and Dvl3 in myotubes, relevant as Dvl2 can affect satellite cell polarity and migration downstream of Wnt7a/Fzd7 74. TAZ S89A downregulates *Fzd6* and *Fzd7*, and *Lgr4, Lgr5,* and *Lgr6*, important Wnt receptors in developmental and regenerative myogenesis 75, 76, suggesting that TAZ desensitize cells to ligands that bind these receptors. For example, quiescent satellite cells express high levels of *Fzd7* but not Taz or Yap, but during activation, the increased abundance/activity of Yap/Taz could be involved in the down‐regulation of *Fdz7*.

Additionally, TAZ S89A increases expression of *Wnt4,* decreases *Sox9* expression and alters expression of secreted Wnt‐associated proteins and genes encoding the structurally related proteins *Wisp1, Wisp2, Igfbp4, Cyr61,* and *Bmp4* 49. Many of these genes have been implicated in regulation of developmental myogenesis, satellite cell function and muscle fibers. For example, over‐expression of Wnt4 increased *Pax7* and *MyoD1* expression in chick embryos 77. Bmp4 promotes satellite cell proliferation but inhibits differentiation 78.

Interestingly, Tead4 bound TAZ and YAP in proliferating myoblasts but not in mature multinucleated myotubes. Tead4 increases dramatically as myoblasts enter myogenic differentiation and levels are then maintained. Thus the TAZ‐Tead4 interaction identified in proliferating myoblasts by proteomics is likely maintained during induction and the early phases of myogenic differentiation, not assayed by proteomics. When Tead4 was knocked down, myogenic differentiation was suppressed, which recovered to controls levels when TAZ S98A was also expressed. However, TAZ S89A could no longer enhance entry into the differentiation programme and augment fusion when Tead4 levels were reduced. Thus, the role of TAZ in inducing and promoting myogenic differentiation is linked to interaction with Tead4 37, 56.

## Conclusion

In conclusion, in the skeletal myogenic lineage we demonstrate similar roles of TAZ and YAP in promoting myoblast proliferation. However, during the later stages of myogenesis, TAZ switches toward influencing satellite cell fate by promoting myogenic differentiation, in a process involving Tead4.

## Author Contributions

C.S.: conception and design, collection and/or assembly of data, data analysis and interpretation, manuscript writing; V.D.M., A.M., A.v.K., E.C.‐D., and D.M.: collection and/or assembly of data, data analysis and interpretation; H.P.O.Q., A.G.‐M., and A.A.B.: collection and/or assembly of data; A.M.T.: conception and design; N.V.: data analysis and interpretation; H.W. and P.S.Z.: conception and design, data analysis and interpretation, manuscript writing.

## Disclosure of Potential Conflicts of Interest

The authors indicate no potential conflicts of interest.

## Supporting information

Supporting Information Table 1.

Supporting Information Table 2.
